# Long-Term Exercise Interventions for Reducing Drug Craving in People with Drug Use Disorder: A Systematic Review and Meta-Analysis

**DOI:** 10.3390/bs15091272

**Published:** 2025-09-18

**Authors:** Xiang Chen, Yuanyuan Jia, Ping Hong, Tingting Sun, Xiaosheng Dong, Jinghua Qian, Junwei Qian, Xiao Hou

**Affiliations:** 1 School of Sport Science, Beijing Sport University, Beijing 100084, China; chenx@bsu.edu.cn (X.C.); jiayy@bsu.edu.cn (Y.J.); 2 Beijing Sport University, Beijing 100084, China; hongping73@bsu.edu.cn; 3 Key Laboratory of Sports and Physical Health Ministry of Education, Beijing Sport University, Beijing 100084, China; 2471@bsu.edu.cn; 4 Department of Social Medicine and Health Management, School of Public Health, Cheeloo College of Medicine, Shandong University, Jinan 250100, China; dxiaosheng@hotmail.com; 5 National Health Commission of China Key Lab of Health Economics and Policy Research (Shandong University), Jinan 250100, China; 6 Center for Health Management and Policy Research, Shandong University (Shandong Provincial Key New Think Tank), Jinan 250100, China; 7 Institute of Health and Elderly Care, Shandong University, Jinan 250100, China; 8 Key Laboratory of Exercise Rehabilitation Science of the Ministry of Education, Beijing Sport University, Beijing 100084, China; qianjinghua@bsu.edu.cn; 9 School of Sport Medicine and Rehabilitation, Beijing Sport University, Beijing 100084, China; 10 Department of Physical Education, Peking University, Beijing 100871, China

**Keywords:** drug use disorder, drug craving, exercise

## Abstract

Exercise is a promising intervention for reducing drug craving, but recent studies have shown inconsistent effects. This meta-analysis aims to evaluate the effect of exercise interventions on drug craving and identify the key exercise factors that affect its effectiveness. The literature was searched in four English databases. Two authors independently performed literature screening, data extraction, and quality assessment. Subgroup, sensitivity, and meta-regression analyses were conducted to explore potential heterogeneity. The results demonstrated that exercise (SMD = −0.74, 95% CI: −0.91, −0.58; *p* < 0.00001) was more effective than control groups in reducing drug craving among people with drug use disorder. Subgroup analyses demonstrated that aerobic (SMD = −0.79, 95% CI: −1.03, −0.54; *p* < 0.00001), multi-component (SMD = −0.96, 95% CI: −1.73, −0.18; *p* = 0.02), and mind–body exercise (SMD = −0.57, 95% CI: −0.88, −0.26; *p* = 0.0003) could significantly reduce drug craving, while resistance exercise (SMD = −0.59, 95% CI: −1.33, 0.16; *p* = 0.12) could not. Moreover, moderate (SMD = −0.77, 95% CI: −0.95, −0.58; *p* < 0.00001) and high-intensity exercise (SMD = −0.63, 95% CI: −1.08, −0.19; *p* = 0.006) were effective in reducing drug craving. In addition, regardless of intervention period, single-session duration, and weekly frequency, exercise could significantly reduce drug craving. This study indicates that exercise effectively reduces drug craving, with type and intensity as key factors affecting the effectiveness.

## 1. Introduction

Drug use disorder is a chronic, relapsing brain disease characterized by compulsive drug-seeking behavior in which individuals continue to use drugs despite harmful consequences such as intoxication and withdrawal (J. F. Liu & Li, 2018; Navarrete et al., 2021). This disease affects a large segment of the global population and represents a significant public health challenge. The World Drug Report 2024 estimated that 64 million people worldwide were affected by drug use disorder in 2022 (UNODC, 2024). The consequences of drug use disorder extend beyond the psychological and physiological harm, also incurring substantial economic costs.

Physiologically, individuals with drug use disorder often suffer from a range of cardiovascular issues (Restrepo et al., 2009). For instance, one study has shown that approximately 25% of nonfatal myocardial infarctions in adults aged 18–45 are associated with cocaine use (Qureshi et al., 2001). Psychologically, individuals with drug use disorder are at a higher risk for mental health disorders, especially depression and anxiety (Jiang et al., 2021). It has been demonstrated that 80% of individuals with drug use disorder experience depression (Tirado Muñoz et al., 2018), and their risk of anxiety disorders is elevated 2.1-fold relative to the general population (Lai et al., 2015). Furthermore, the treatment of drug use disorder imposes a substantial economic burden on healthcare systems. Due to the high cost of addiction treatment, only approximately one in eleven individuals with a drug use disorder receives drug treatment globally (UNODC, 2024). For instance, in Israel, the average monthly healthcare cost for individuals with opioid use disorder was estimated at USD 1102, exceeding USD 13,000 annually (Miron et al., 2022). Similarly, in Australia, the annual healthcare cost per heroin-dependent person was AUD 10,055 (Hall et al., 2024). These costs impose significant financial stress on individuals, families, and society. Given that, it is necessary to develop effective treatment strategies for individuals with drug use disorder to reduce drug craving.

Drug craving is a key diagnostic criterion for drug use disorder (American Psychiatric Association, 2013) and plays a central role in sustaining addiction by driving drug-seeking behaviors (Sayette, 2016). The American Psychiatric Association (APA) defines drug craving as a strong desire or urge to use drugs, which can occur at any time (American Psychiatric Association, 2013). It is also recognized as a significant factor contributing to relapse (Moreno-Rius & Miquel, 2017), which severely affects the treatment of drug use disorder (UNODC, 2024). It has been indicated that a 1-point increase on a 0–100 craving scale raises the likelihood of methamphetamine (MA) use by 0.38% (Galloway & Singleton, 2009). In addition, nearly 60% of patients relapse within a year following conventional interventions (Agosti et al., 2012; Ramo & Brown, 2008). Thus, effectively suppressing craving is critical to improving drug use disorder treatment outcomes.

Current intervention strategies for reducing drug cravings among addicts usually rely on pharmacological interventions, but this strategy still faces several challenges, such as high costs, mandatory administration requirements, and adverse side effects (Gupta et al., 2021). These issues underscore the need for complementary treatments that are more accessible, tolerable, and cost-effective. Recently, exercise has emerged as a promising adjunctive therapy for drug use disorder due to its positive effects on cognitive function (Zheng et al., 2024). Specifically, inhibitory control deficit is regarded as a core marker of drug use disorder (Dawe et al., 2004; Kalivas & Volkow, 2005) and has been shown to be negatively related to drug craving (Li et al., 2025). Exercise has been found to enhance inhibitory control among individuals with MA dependence (Menglu et al., 2021), which may help reduce their drug craving. Wang et al. also have reported a significantly reduced drug craving after a 12-week aerobic exercise compared to baseline levels in MA-dependent patients (D. Wang et al., 2017). However, another study has yielded no significant reduction in drug craving following either aerobic or resistance exercise in MA-dependent individuals (Y. Lu et al., 2021). This discrepancy emphasizes the necessity for further investigation into the potential factors influencing the effectiveness of exercise and synthesizing the effects of exercise on drug craving.

In summary, current evidence regarding the therapeutic effects of exercise on drug craving reduction remains inconclusive, and the effect of exercise may vary depending on factors such as type, intensity, intervention period, single-session duration, and weekly intervention frequency (Dong et al., 2024; Y. Lu et al., 2021). This study aims to conduct a meta-analysis to systematically evaluate the effects of exercise interventions on reducing drug craving, with subgroup analyses across different exercise types, intensities, intervention periods, single-session durations, and weekly intervention frequencies. The findings are expected to provide an evidence-based framework for optimizing exercise prescriptions in addiction rehabilitation.

## 2. Methods

### 2.1. Search Strategy

This systematic review and meta-analysis was based on the Preferred Reporting Items for Systematic Reviews and Meta-Analyses (PRISMA) guidelines and registered in the International Prospective Register of Systematic Reviews (PROSPERO; http://www.crd.york.ac.uk/PROSPERO; accessed on 2 April 2025) under the registration number CRD 42024567127. The authors systematically searched four databases (PubMed, Web of Science, Embase, and the Cochrane Library) for records published from inception to 2 August 2025, using the following search terms: (“Exercis*” OR “Physical exercis*” OR “Train*”OR “Aerobic exercis*” OR “Isometric exercis*” OR “Acute exercis*” OR “Exercise train*” OR “Physical activit*” OR “Resistance train*” OR “Weight train*” OR “Strength train*” OR “Sport*” OR “Tai chi*” OR “danc*” OR “bicycl*” OR “yoga*” OR “Tai-ji*” OR “Taijiquan” OR “Qi Gong”) AND (“Substance-Related Disorders” OR “Drug Use Disorder” OR “Substance Abuse” OR “Substance Dependence” OR “Drug Addiction” OR “Drug Abuse” OR “Substance use disorder” OR “Drug” OR “Methamphetamine” OR “Cocaine” OR “Cannabis” OR “Heroin” OR “Amphetamine” OR “opium” OR “opioids” OR “cannabinoids” OR “Ecstasy” OR “morphine” OR “marijuana”) AND (“craving” OR “desire”) AND (“Randomized controlled trial” OR “Randomized” OR “Controlled” OR “Trial”). Additionally, manual screening of reference lists was performed to ensure literature saturation.

### 2.2. Study Selection

EndNote 20 was used to remove duplicate records. Two authors (X.C. and Y.J.) independently screened titles, abstracts, and full texts to identify studies meeting the inclusion criteria. Disagreements in study selection were resolved through discussion with a third author (X.H.) when necessary.

Inclusion and exclusion criteria adhered to the PICOS framework:

#### 2.2.1. Inclusion Criteria

(1) Participants (P): Adults aged 18–65 diagnosed with drug use disorder;
(2) Intervention (I): Experimental groups received structured and long-term (≥4 weeks) exercise interventions;
(3) Comparison (C): Control groups received routine care (e.g., health education) or no intervention;
(4) Outcome (O): Drug craving was quantified using validated scales (e.g., Visual Analogue Scale (Sayette et al., 2000), Amphetamine Craving Questionnaire (James et al., 2004));
(5) Study design (S): Randomized controlled trials (RCTs).

#### 2.2.2. Exclusion Criteria

(1) Non-human studies, reviews, conference abstracts, or case reports;
(2) Acute exercise interventions (<4 weeks);
(3) Combined interventions (exercise co-administered with other therapies);
(4) Insufficient data for effect size calculation;
(5) Non-English studies or unavailable full texts.

### 2.3. Data Extraction

Two authors (X.C. and Y.J.) independently extracted data using a standardized form, which included (1) study characteristics (first author, publication year); (2) participant demographics (sample size, country); (3) intervention parameters (exercise type, intensity, period, session duration, frequency); (4) drug types; and (5) outcome measures (craving assessment scales). Drug craving outcomes were recorded as mean ± standard deviation (M ± SD). If the standard deviation (SD) was not directly reported in the original study, it could be estimated using standard error (SE), confidence interval (CI), *p*-values, or *t*-statistics, in accordance with the Cochrane Collaboration Handbook recommendations. Data from figures were extracted using GetData Graph Digitizer 2.26. Any discrepancies in data extraction were resolved through discussion with a third author (X.H.) when necessary.

### 2.4. Quality Assessment

Two authors (X.C. and Y.J.) independently evaluated study quality using the Cochrane Risk of Bias Tool (Higgins et al., 2011). Any discrepancies in quality assessment were resolved through discussion with a third author (X.H.) when necessary. The bias evaluation covered seven domains: random sequence generation, allocation concealment, blinding of participants and personnel, blinding of outcome assessment, incomplete outcome data, selective reporting, and other bias.

### 2.5. Data Analysis

Given potential baseline heterogeneity across studies, between-group comparisons using post-intervention M ± SD could induce bias. Hence, the mean_of changes_ ± SD_of changes_ was calculated from baseline and post-intervention data. The calculation formula is based on the Cochrane Handbook (Higgins et al., 2024): Mean_of changes_ = Mean_post-intervention_ − Mean_post-intervention_; SD_of changes_ = √(SD_pre-intervention_^2^ + SD_post-intervention_^2^ − 2 × 0.6 × SD_pre-intervention_ × SD_post-intervention_).

Meta-analyses were conducted using RevMan 5.4 and Stata 15.1 software. Standardized mean differences (SMDs) with a 95% confidence interval (CI) were calculated to account for heterogeneity in measurement instruments and intervention protocols, with statistical significance defined as α = 0.05. Heterogeneity was quantified using *I*^2^: studies with *I*^2^ ≤ 50% were considered to exhibit low heterogeneity and were analyzed using fixed-effect models; those with *I*^2^ > 50% indicated substantial heterogeneity and were analyzed using random-effects models.

Subgroup analyses were conducted based on exercise type, intensity, intervention period, single-session duration, and weekly intervention frequency to investigate differential efficacy across exercise parameters and identify heterogeneity sources. Referring to previous studies (X. Huang et al., 2022), the types of exercise were classified into the following categories: (1) aerobic exercise (AE); (2) resistance exercise (RE); (3) multi-component exercise (ME); (4) mind–body exercise (MBE); (5) other types. The exercise intensity was classified into three categories: (1) Low; (2) Moderate; (3) High. The intensities of exercise were based on heart rate, percentage of the maximum number of repetitions at one time (%1RM), number of repetitions (RM), or rating of perceived exertion (PRE) that was provided in the included studies. For the intervention period (Dong et al., 2024), it was classified as (1) short (≤3 months); (2) medium (3~12 months); or (3) long (≥12 months). The single-session intervention duration (Dong et al., 2024) was classified into three levels: (1) short (≤30 min/time); (2) medium (30~60 min/time); and (3) long (≥60 min/time). The weekly intervention frequency (Dong et al., 2024) was classified into three categories: (1) short (≤3 times/week); (2) medium (4~5 times/week); and (3) high (6~7 times/week).

This study conducted a sensitivity analysis by excluding each study one by one. Meanwhile, the meta-regression analysis was performed to further explore the sources of heterogeneity, based on the publication year, the baseline level of the outcomes, the method of intervention used in the control group, the sample size, and the type of drug. Additionally, funnel plots, Begg’s test, and Egger’s test were adopted to evaluate the publication bias of the included studies.

## 3. Results

### 3.1. Characteristics of the Included Studies

**Figure 1:**
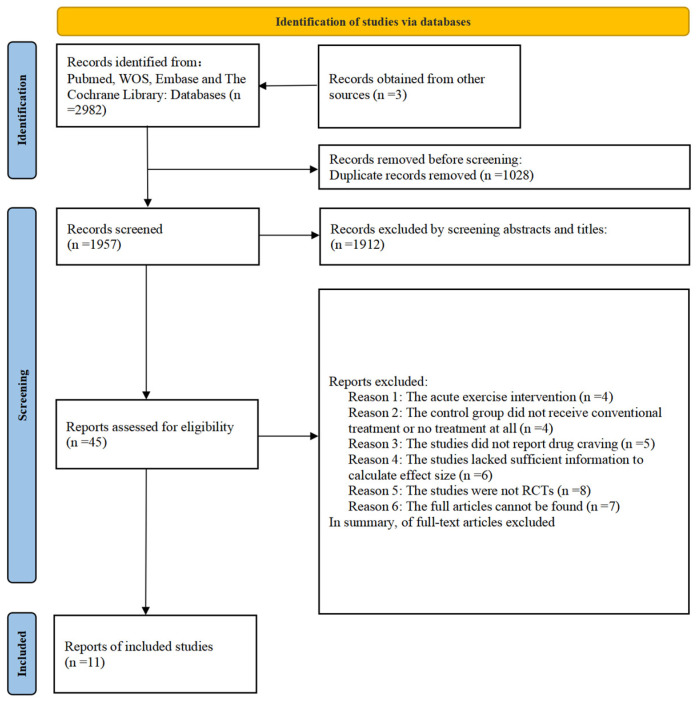
The flow diagram of the search procedure.

The database searches initially identified 2982 records. After removing 1028 duplicates and excluding 1912 studies based on title and abstract screening, 45 studies remained for full-text assessment. Finally, 11 eligible RCTs involving 636 participants were included in this meta-analysis (Chen et al., 2021; De la Garza et al., 2016; X.-x. Liu & Wang, 2023; X. X. Liu et al., 2024; Y. Lu et al., 2021; Tan et al., 2023; D. Wang et al., 2017; J. Wang et al., 2021; M. Wang et al., 2024; Zhang & Zhu, 2020; Zhu et al., 2022). The search procedure is presented in Figure 1.

The detailed characteristics of the 11 included studies are summarized in Table 1. Ten studies were conducted in China, and one study was conducted in the United States, with publication years ranging from 2016 to 2024. In the exercise type subgroup, seven studies used AE (Chen et al., 2021; De la Garza et al., 2016; X.-x. Liu & Wang, 2023; X. X. Liu et al., 2024; Y. Lu et al., 2021; D. Wang et al., 2017; Zhu et al., 2022), one study used RE (Y. Lu et al., 2021), two studies used ME (Tan et al., 2023; J. Wang et al., 2021), two studies used MBE (M. Wang et al., 2024; Zhang & Zhu, 2020), and no studies used other exercise types. For exercise intensity, 1 study conducted a low-intensity protocol (De la Garza et al., 2016), 10 studies conducted a moderate-intensity protocol (Chen et al., 2021; De la Garza et al., 2016; X.-x. Liu & Wang, 2023; X. X. Liu et al., 2024; Y. Lu et al., 2021; D. Wang et al., 2017; J. Wang et al., 2021; M. Wang et al., 2024; Zhang & Zhu, 2020; Zhu et al., 2022), 2 studies conducted a high-intensity protocol (Chen et al., 2021; Tan et al., 2023), and 1 study did not report precise intensity parameters (Y. Lu et al., 2021). For the intervention period, nine studies designed short-period exercise intervention (Chen et al., 2021; De la Garza et al., 2016; X.-x. Liu & Wang, 2023; X. X. Liu et al., 2024; Y. Lu et al., 2021; D. Wang et al., 2017; J. Wang et al., 2021; M. Wang et al., 2024; Zhu et al., 2022), two studies designed medium-period exercise intervention (Tan et al., 2023; Zhang & Zhu, 2020), and no studies designed long-term periods. For the single-session duration, four studies involved short-duration interventions (Chen et al., 2021; De la Garza et al., 2016; D. Wang et al., 2017; Zhang & Zhu, 2020), two studies involved medium-duration interventions (Y. Lu et al., 2021; Zhu et al., 2022), and five studies involved long-duration interventions (X.-x. Liu & Wang, 2023; X. X. Liu et al., 2024; Tan et al., 2023; J. Wang et al., 2021; M. Wang et al., 2024). One resistance training did not report single-session duration (Y. Lu et al., 2021). Regarding the weekly intervention frequency, five studies adopted short-frequency intervention (Chen et al., 2021; De la Garza et al., 2016; X. X. Liu et al., 2024; Y. Lu et al., 2021; D. Wang et al., 2017), six studies adopted medium-frequency intervention (X.-x. Liu & Wang, 2023; Tan et al., 2023; J. Wang et al., 2021; M. Wang et al., 2024; Zhang & Zhu, 2020; Zhu et al., 2022), and no studies adopted high-frequency exercise intervention. All studies adopted validated quantitative scales to assess drug craving levels.

### 3.2. Included Literature Quality

**Figure 2:**
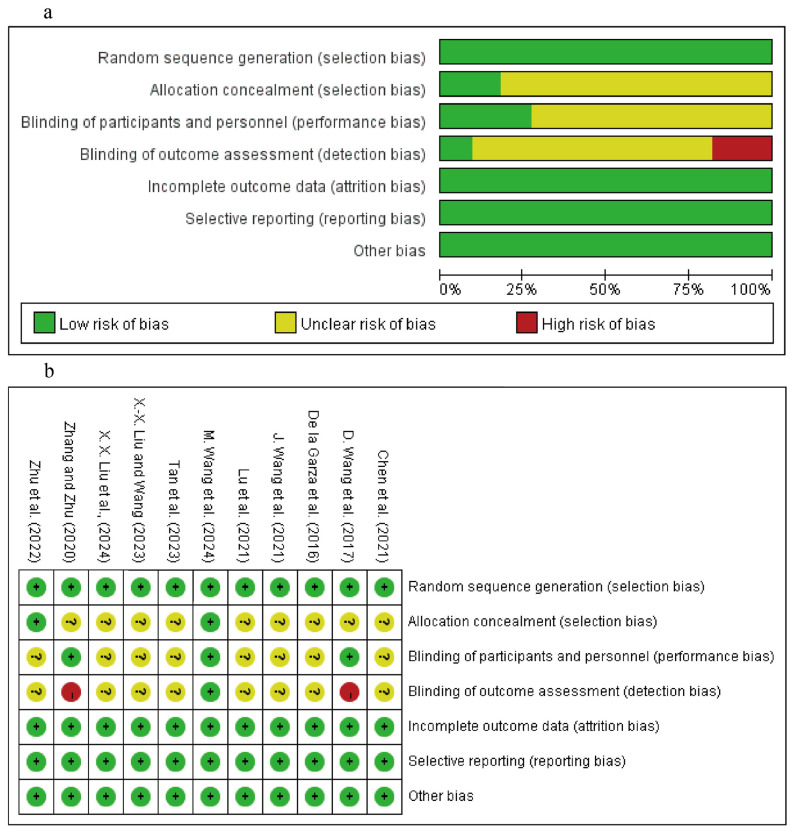
Risk of bias assessment. (**a**) Risk of bias graph; (**b**) Risk of bias summary. Abbreviations: “+”: High risk; “-”: Low risk; “?”: Unclear risk. (Chen et al., 2021; D. Wang et al., 2017; De la Garza et al., 2016; J. Wang et al., 2021; Y. Lu et al., 2021; M. Wang et al., 2024; Tan et al., 2023; X.-x. Liu & Wang, 2023; X. X. Liu et al., 2024; Zhang & Zhu, 2020; Zhu et al., 2022).

Based on the risk of bias assessment, four studies were rated as high-quality (D. Wang et al., 2017; M. Wang et al., 2024; Zhang & Zhu, 2020; Zhu et al., 2022) and seven studies were rated as moderate-quality (Chen et al., 2021; De la Garza et al., 2016; X.-x. Liu & Wang, 2023; X. X. Liu et al., 2024; Y. Lu et al., 2021; Tan et al., 2023; J. Wang et al., 2021). The primary sources of bias were related to allocation concealment, blinding of participants and personnel, and blinding of outcome assessment. Detailed risk of bias profiles are presented in Figure 2.

### 3.3. Effects of Exercise on Drug Craving

**Figure 3:**
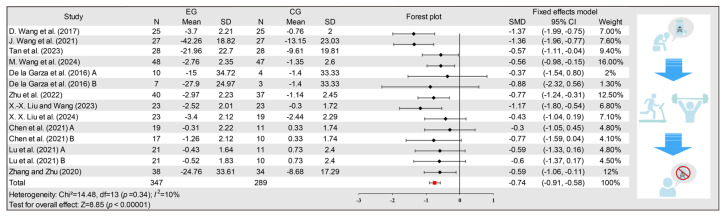
Forest plot of the effect of exercise on drug craving. Abbreviations: EG: experimental group; CG: control group; N: sample size; SD: standard deviation; SMD: standardized mean differences; 95% CI: 95% confidence intervals. (D. Wang et al., 2017; J. Wang et al., 2021; Tan et al., 2023; M. Wang et al., 2024; De la Garza et al., 2016; Zhu et al., 2022; X.-x. Liu & Wang, 2023; X. X. Liu et al., 2024; Chen et al., 2021; Y. Lu et al., 2021; Zhang & Zhu, 2020).

A total of 14 data points from 11 studies reported the effect of exercise on drug craving among individuals with drug use disorder. Due to low heterogeneity (*I*^2^ = 10%), a fixed-effect model was used for the meta-analysis. Overall, as shown in Figure 3, The pooled analysis of 636 participants presented that exercise interventions significantly reduced drug craving compared with the control group (SMD = −0.74, 95% CI: −0.91, −0.58; *p* < 0.00001).

### 3.4. Subgroup Analysis

**Figure 4:**
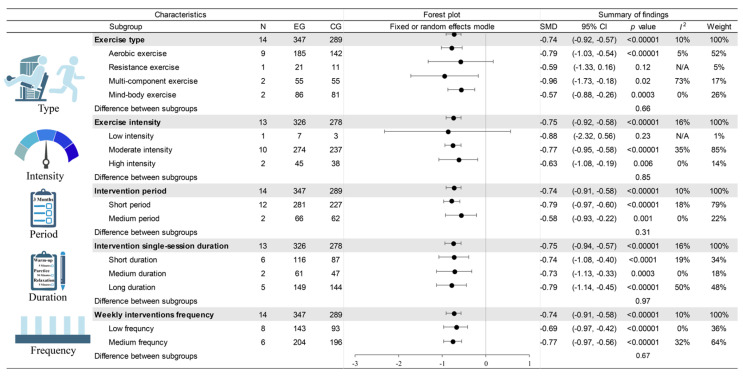
Forest plot of the effect of exercise on drug craving for different subgroups. Abbreviations: N: Number of data points in randomized controlled trials; EG: experimental group; CG: control group; SD: standard deviation; SMD: standardized mean differences; 95% CI: 95% confidence interval.

Figure 4 presents the subgroup analysis results about the effect of exercise on drug craving based on exercise type, exercise intensity, intervention period, single-session intervention duration, and weekly intervention frequency.

There was no significant difference in the effects of different exercise types on drug craving (Chi^2^ = 1.61, df = 3, *p* = 0.66). However, compared with the control group, AE (SMD = −0.79, 95% CI: −1.03, −0.54; *p* < 0.00001), ME (SMD = −0.96, 95% CI: −1.73, −0.18; *p* = 0.02), and MBE (SMD = −0.57, 95% CI: −0.88, −0.26; *p* = 0.0003) could significantly reduce drug craving. No significant difference was observed between the RE group (SMD = −0.59, 95% CI: −1.33, 0.16: *p* = 0.12) and the control group.

Compared to the control group, the moderate-intensity (SMD = −0.77, 95% CI: −0.95, −0.58; *p* < 0.00001) and high-intensity exercise protocols (SMD = −0.63, 95% CI: −1.08, −0.19; *p* = 0.006) showed a significant drug craving reduction, while the low-intensity (SMD = −0.88, 95% CI: −2.32, 0.56; *p* = 0.23) exercise protocol did not show significant effects. However, no significant difference was observed in the effects of different exercise intensities on drug craving (Chi^2^ = 0.32, df = 2, *p* = 0.85).

For intervention periods, there was no significant difference between the short- and medium-period interventions in the effects on drug craving (Chi^2^ = 1.02, df = 1, *p* = 0.31). To be specific, compared with the control group, both short-period (SMD = −0.79, 95% CI: −0.97, −0.60; *p* < 0.00001) and medium-period interventions (SMD = −0.58, 95% CI: −0.93, −0.22; *p* = 0.001) had significant effects on drug craving reduction.

Furthermore, there was no significant difference in the effects of different single-session intervention durations (Chi^2^ = 0.07, df = 2, *p* = 0.97). Specifically, whether short-duration (SMD = −0.74, 95% CI: −1.08, −0.40; *p <* 0.0001), medium-duration (SMD = −0.73, 95% CI: −1.13, −0.33; *p* = 0.0003), or long-duration (SMD = −0.79, 95% CI: −1.14, −0.45; *p* < 0.00001), all single-session duration interventions could significantly reduce drug craving compared with the control group.

Regarding weekly intervention frequency, no significant difference was observed between low- and medium-frequency interventions (Chi^2^ = 0.19, df = 1, *p* = 0.67). To be specific, both low-frequency (SMD = −0.69, 95% CI: −0.97, −0.42; *p* < 0.00001) and medium-frequency interventions (SMD = −0.77, 95% CI: −0.97, −0.56; *p* < 0.00001) could significantly reduce drug craving, compared with the control group.

### 3.5. Publication Bias

**Figure 5:**
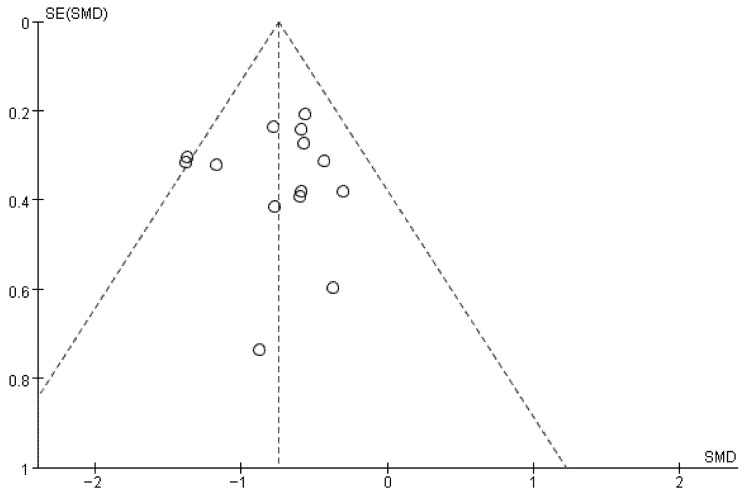
Funnel plots.

Visual inspection of the funnel plot (Figure 5) showed no notable asymmetry. In addition, no significant publication bias was found in the Begg’s test (*p* = 0.66) and Egger’s test (*p* = 0.84). Therefore, the meta-analyses suggest a low likelihood of publication bias among the included studies.

### 3.6. Meta-Regression Analysis

A meta-regression analysis was conducted to explore potential influencing factors of effect size, including the publication year, the baseline level of the outcomes, the method of intervention used in the control group, the sample size, and the type of drug. The results indicated that there were no significant confounding factors. Detailed results are shown in Table 2.

## 4. Discussion

This meta-analysis demonstrated that exercises could significantly reduce drug craving among individuals with drug use disorder. For exercise type, AE, ME, and MBE showed significant effects on drug craving reduction compared with the control group, while RE did not demonstrate a significant effect. Furthermore, subgroup analyses of exercise intensity demonstrated that moderate- and high-intensity exercise interventions were effective in reducing drug craving, while low-intensity exercise interventions could not. Notably, exercise could significantly reduce drug craving among individuals with drug use disorder, irrespective of intervention period, single-session duration, or weekly frequency.

Our results that exercise could significantly reduce drug craving might be explained by the neurobiological foundations. Specifically, chronic drug use leads to a reduction in the number of dopamine receptors and transporters at the micro level (McCann et al., 2008; Volkow & Morales, 2015) and to structural damage and decreased metabolic activity in the prefrontal cortex at the macro level (Goldstein & Volkow, 2002, 2011). These neurobiological changes result in impaired inhibitory control and working memory dysfunction, which may contribute to poor decision-making, compulsive drug-seeking behaviors, and reduced ability to suppress drug cravings (Chambers et al., 2009; Goldstein & Volkow, 2011; Volkow et al., 2016). Collectively, these micro- and macro-level impairments compromise the brain’s ability to regulate drug craving. Exercise may suppress the pathological reward circuit by reversing dopamine system impairments. The mesolimbic dopamine circuit, commonly known as the brain’s reward circuit (Heshmati & Russo, 2015), is altered by chronic drug abuse, resulting in pathological circuits that not only enhance the responsiveness to drug cues but also reduce the sensitivity to natural rewards, thereby contributing to drug craving (Volkow & Morales, 2015). Exercise can promote the release of endogenous dopamine and endorphins, which may substitute for drug-induced stimulation and produce a natural physiological reward (Cameron et al., 2017). An animal-based study has found that 6 weeks of AE can increase striatal dopamine D2 receptor density (Robison et al., 2018). A human-based trial has also shown that 8 weeks of AE can elevate striatal D2/D3 receptor availability compared with the control group in MA users (Robertson et al., 2016). These findings suggest that exercise may help restore the damaged reward circuit by promoting dopamine release and receptor regeneration (J. Huang et al., 2019). At the neurobiological level, this recovery supports the prefrontal cortex’s regulatory control over impulses and cravings, thereby contributing to reduced drug craving. However, the explanation of this mechanism requires further empirical testing in future clinical trials.

Based on exercise type subgroups, our meta-analysis has found that AE, ME, and MBE can significantly reduce drug craving, while RE cannot. For AE, previous studies have demonstrated that 12 weeks of AE can improve inhibitory control and working memory (Chen et al., 2021; D. Wang et al., 2017), thereby enhancing the ability to suppress drug cravings. For ME, the combination of aerobic and resistance training not only provides the benefits of AE in reducing drug cravings but also improves exercise adherence (Chang et al., 2023), thereby optimizing overall intervention efficacy. For the MBE subgroup, the included studies used Tai Chi, a specialized practice integrating meditation with moderate AE, as the intervention protocol. It has been indicated that Tai Chi can increase oxygenated hemoglobin concentration (X. Lu et al., 2016) and activate the prefrontal cortex (Wu et al., 2018), suggesting that it may enhance activity in this brain region, improve inhibitory control, and consequently reduce drug craving (He et al., 2021). In contrast, RE showed no significant effect on craving reduction compared to the control group. This may be due to the lower release of dopamine and endorphins during RE (Shimojo et al., 2019), which may fail to sufficiently activate reward pathways. This, in turn, may lead to training-related boredom, decreased adherence, and mental fatigue (Segura-García et al., 2010). Furthermore, brain-derived neurotrophic factor (BDNF) plays a pivotal role in modulating brain signaling and synaptic plasticity (Kowiański et al., 2018). In drug-free conditions, cue-induced cocaine seeking is suppressed by endogenous BDNF in the nucleus accumbens core (NAcore) (Bobadilla et al., 2019). BDNF can be stimulated by exercise, thereby enhancing plasticity and modifying damaged neural networks. However, a systematic review has shown that RE does not significantly affect basal peripheral BDNF levels (Knaepen et al., 2010). These findings partly support the notion that RE may not significantly reduce drug craving, a hypothesis that requires further empirical investigation.

The exercise intensity-based subgroup analysis showed that moderate- and high-intensity programs significantly reduced drug cravings, while low-intensity programs did not significantly reduce drug cravings. The craving-reducing effects of moderate- and high-intensity exercise may be explained by the elevation of endocannabinoid levels (Brellenthin et al., 2017; Raichlen et al., 2012; Sparling et al., 2003). Endocannabinoids are a class of naturally occurring lipid-based neurotransmitters in the human body that exert effects similar to those of cannabinoids. They can synergize with the dopamine system to activate the body’s natural reward pathways (Gupta et al., 2024), thereby reducing the rewarding effects induced by exogenous addictive drugs. In contrast, low-intensity exercise may not sufficiently elevate endocannabinoid levels (Raichlen et al., 2012). Additionally, the low-intensity subgroup included only one study, which may have contributed to bias in the results.

Subgroup analyses of intervention period, single-session duration, and weekly intervention frequency show that exercise interventions can significantly reduce drug craving, regardless of intervention period, single-session duration, or weekly frequency. This suggests that, within the intervention period, duration and weekly frequency ranges in included studies, exercise-induced reductions in drug craving demonstrate considerable consistency and stability. Despite our predefined subgroups for long-term interventions (≥12 months) and high-frequency interventions (6–7 times/week) in this meta-analysis, no studies met these criteria. This gap may reflect current limitations in this field: structured exercise interventions with extended periods or high frequencies remain scarce. This scarcity may be due to feasibility challenges, such as participant adherence and intervention costs. As a result, this absence limits the ability to further investigate the effects of different exercise periods and frequencies on reducing drug use disorder, particularly long-term and high-frequency exercise interventions.

This meta-analysis provides a more comprehensive and more accurate systematic evaluation of the effects of different exercise types, intensities, and intervention periods, single-session durations, and weekly intervention frequencies on drug craving reduction among individuals with drug use disorder. However, several limitations should be noted. First, our risk of bias assessment revealed that many included studies had unclear or high risk of bias in allocation concealment and blinding. Inadequate allocation concealment may give rise to selection bias, thereby undermining the baseline comparability of intervention groups. Similarly, the absence of blinding for participants and outcome assessors can heighten the risk of performance and detection bias, a concern that is particularly salient for drug craving outcomes relying predominantly on self-reported measures. Such biases could potentially overestimate or underestimate the true effects of exercise interventions. Therefore, the present findings should be interpreted with caution. Future randomized controlled trials should adopt rigorous blinding procedures and ensure proper allocation concealment to improve the internal validity and reliability of the evidence base. Second, during data extraction, some studies did not report M ± SD directly, which required data to be derived through formula-based or figure-based conversions. This process may have introduced inaccuracies and resulted in some data loss. Third, this meta-analysis may be subject to language bias, as only English-language studies were included. Additionally, given differences in cultural attitudes toward addiction, healthcare systems, and social support policies, the fact that 10 of the 11 studies included in this meta-analysis were conducted in China may limit the global applicability of the findings. In the future, cross-cultural studies are needed to validate the universality of these results. Finally, the relatively small number of included studies limits the applicability of our findings primarily to cocaine, methamphetamine, and amphetamine use disorders. Moreover, this limited sample size may also have affected the subgroup analyses and hindered exploration of potential sources of heterogeneity, such as participant characteristics (e.g., addiction duration, severity, and comorbidities), potentially introducing bias into the results.

## 5. Conclusions

Exercise demonstrates a significant effect in reducing drug craving among individuals with a drug use disorder. The type and intensity of exercise, rather than intervention period, single-session duration, or weekly intervention frequency, are likely key influencing factors in the success of exercise intervention in reducing drug craving. Therefore, when performing exercise interventions for drug craving, it is recommended that exercise programs be designed for moderate- or high-intensity exercises in the form of AE, ME, or MBE.

## Figures and Tables

**Table 1:** Characteristics of included studies.

Author (Year)	Country	Sample Size	Experimental Interventions					Control Interventions	Drug Information	Outcome
			Exercise Type	Exercise Intensity	Intervention Period	Intervention Single-Session Duration	Weekly Intervention Frequency			
D. Wang et al. (2017)	CHINA	EG: n = 25 CG: n = 25	Aerobic exercise (AE)	Moderate	12 weeks	30 min	3 times	Routine care	Methamphetamine	VAS
J. Wang et al. (2021)	CHINA	EG: n = 27 CG: n = 27	Resistance and aerobic exercise (ME)	Moderate	8 weeks	60 min	5 times	Safety and health education	Methamphetamine	VR-VAS
Tan et al. (2023)	CHINA	EG: n = 28 CG: n = 28	High-intensity interval training (ME)	High	8 months	60 min	4 times	Routine rehabilitation therapy	Methamphetamine	VAS
M. Wang et al. (2024)	CHINA	EG: n = 48 CG: n = 47	Tai chi (MBE)	Moderate	3 months	60 min (per training session 30 min)	5 times (two training sessions per time)	Traditional addiction treatments	Methamphetamine	VAS
De la Garza et al. (2016) A	AMERICA	EG: n = 10 CG: n = 4	Running (AE)	Moderate	4 weeks	30 min	3 times	Sitting	Cocaine	VAS
De la Garza et al. (2016) B	AMERICA	EG: n = 7 CG: n = 3	Walking (AE)	Light	4 weeks	30 min	3 times	Sitting	Cocaine	VAS
Zhu et al. (2022)	CHINA	EG: n = 40 CG: n = 37	Aerobic gymnastics (AE)	Moderate	3 months	36 min (30 min in 1st month)	5 times	Routine care	Methamphetamine	VAS
X.-x. Liu and Wang (2023)	CHINA	EG: n = 23 CG: n = 23	Aerobic exercise (AE)	Moderate	8 weeks	60 min	5 times	Health Education	Methamphetamine	VAS
X. X. Liu et al. (2024)	CHINA	EG: n = 23 CG: n = 19	Aerobic exercise (AE)	Moderate	8 weeks	60 min	3 times	Health Education	Methamphetamine	VAS
Chen et al. (2021) A	CHINA	EG: n = 19 CG: n = 11	Aerobic exercise (AE)	Moderate	12 weeks	30 min	3 times	Drug rehabilitation education and simple manual labor	Methamphetamine	VAS
Chen et al. (2021) B	CHINA	EG: n = 17 CG: n = 10	Aerobic exercise (AE)	High	12 weeks	30 min	3 times	Drug rehabilitation education and simple manual labor	Methamphetamine	VAS
Y. Lu et al. (2021) A	CHINA	EG: n = 21 CG: n = 11	Resistance exercise (RE)	N/A	12 weeks	N/A	3 times	Routine care	Methamphetamine	VAS
Y. Lu et al. (2021) B	CHINA	EG: n = 21 CG: n = 10	Cycling exercise (AE)	Moderate	12 weeks	40 min	3 times	Routine care	Methamphetamine	VAS
Zhang and Zhu (2020)	CHINA	EG: n = 38 CG: n = 34	Tai chi (MBE)	Moderate	6 months	1 time 50 min/ 4 times 20 min	5 times	Routine rehabilitation exercises	Amphetamine	DSQ

Abbreviations: AE: aerobic exercise; RE: resistance exercise; ME: multi-component exercise; MBE: mind–body exercise; EG: experimental group; CG: control group; VAS: visual analog scale; VR-VAS: Virtual Reality-Visual Analog Scale; DSQ: psychological craving.

**Table 2:** Result of meta-regression analysis.

Potential Confounding Factors	*β*	95% CI	*p*
The publication year	0.14	(−0.02, 0.3)	0.07
The baseline level of EG	0	(−0.23, 0.23)	0.99
The baseline level of CG	−0.01	(−0.24, 0.22)	0.94
The method of intervention used in the control group	0.01	(−0.1, 0.12)	0.89
The sample size	0	(−0.01, 0.01)	0.87
The type of drug	−0.67	(−1.53, 0.19)	0.11

Abbreviations: CI: confidence interval; EG: experimental group; CG: control group.

## Data Availability

No new data were created or analyzed in this study.
